# A Personalised and Gamified Digital Decision Aid for Colorectal Cancer Screening: A Randomised Controlled Pilot Study

**DOI:** 10.1002/cam4.71991

**Published:** 2026-05-29

**Authors:** Mattia Celebrin, Asia Filosa, Manuela Anelli, Francesco Brucchi, Paolo La Torraca Vittori, Alessandro Incremona, Mattia Rogledi, Alessandro Summer, Maria Vizzardi, Paola Ghidini, Michela Bregoli, Enri Hoxha, Giovanni Marazza, Alessandra Gorini, Cristina Montomoli

**Affiliations:** ^1^ Unit of Biostatistics and Clinical Epidemiology, Department of Public Health, Experimental and Forensic Medicine University of Pavia Pavia Italy; ^2^ University of Milan Milan Italy; ^3^ Division of General and Endocrine Surgery IRCCS Istituto Auxologico Italiano Milan Italy; ^4^ Department of Brain and Behavioural Sciences University of Pavia Pavia Italy; ^5^ Department of Mathematics and Physics “Niccoló Tartaglia”; Faculty of Mathematical, Physical and Natural Sciences Università Cattolica del Sacro Cuore Brescia Italy; ^6^ Department of Industrial and Information Engineering University of Pavia Pavia Italy; ^7^ School of Physics Trinity College Dublin Ireland; ^8^ Trinity Quantum Alliance Dublin Ireland; ^9^ Department of Public Health and Preventive Medicine ATS Brescia Brescia Italy; ^10^ Department of Clinical Sciences and Community Health (Department of Excellence 2023‐2027) University of Milan Milan Italy; ^11^ Istituti Clinici Scientifici Maugeri IRCCS Milan Italy

**Keywords:** colorectal cancer, decision aid, eHealth, gamification, screening

## Abstract

**Background:**

Colorectal cancer (CRC) is the second leading cause of tumour‐related mortality worldwide, accounting for approximately 903,000 deaths annually. Despite the proven effectiveness of population‐based screening in reducing mortality, participation rates remain suboptimal, hindered by cognitive, psychological, and socioeconomic barriers.

**Objective:**

This study assessed the effectiveness of the PREVenGO smartphone application, a digital decision aid (DA) incorporating personalisation and gamification elements, in supporting informed decision‐making and adherence to CRC screening.

**Methods:**

DA‐CRC is a two‐arm randomised controlled pilot trial. Individuals born in 1973–1974 and invited for the first time to the CRC screening programme by the Local Health Authority of Brescia (Northern Italy) were eligible. Invitation letters included instructions to download the PREVenGO app, which randomised participants into intervention or control arms. The intervention app integrated questionnaires on family history, prevention knowledge, and locus of control (LoC), together with gamified features and rewards, while the control version provided static educational material. Both groups received follow‐up notifications at 3 (T1) and 6 months (T2).

**Results:**

Among those invited, 1% (*n* = 248) downloaded the app and completed onboarding. Follow‐up retention was limited: only 18 participants (7.3%) completed the 3‐month questionnaire (T1) and 1 (0.4%) the 6‐month assessment (T2). The overall faecal occult blood test (FOBT) screening uptake was 86.7% (*n* = 215), with comparable rates in both arms (control: 84.3%, *n* = 102; intervention: 89.0%, *n* = 113; *p* = 0.278). Only 17% (*n* = 43) of participants met all Marteau framework criteria for informed choice, with critical knowledge gaps, particularly regarding overdiagnosis, false positives, and absolute screening benefit.

**Conclusions:**

Preliminary findings suggest that a digital DA with personalised and gamified content may enhance CRC screening uptake and partially support informed choice. Larger, multisite studies are warranted to evaluate scalability, inclusivity, and long‐term effectiveness in population‐based cancer prevention.

**Clinical Registration:**

ClinicalTrials.gov RCT n. NCT07269028. (https://clinicaltrials.gov/study/NCT07269028?term=NCT07269028&rank=1)

## 1 Introduction

Colorectal cancer (CRC) is the second leading cause of cancer‐related death worldwide, with over 900,000 estimated deaths annually [1]. Population‐based screening programmes, typically involving faecal occult blood testing (FOBT) followed by diagnostic colonoscopy in case of a positive result, are among the most effective strategies for secondary prevention, resulting in early diagnosis and reduced mortality. It should be noted that screening approaches vary internationally: in the United States, guidelines recommend either faecal immunochemical testing (FIT) followed by colonoscopy if positive, or upfront colonoscopy, with screening initiation at age 45 [2]. The present study was conducted within the Italian organised screening programme, which uses guaiac faecal occult blood test (FOBT) with colonoscopy for positive results, beginning at age 50. However, despite well‐established efficacy, participation in organised CRC screening remains below the thresholds recommended by clinical guidelines in many regions, including those with robust healthcare systems [3]. Low adherence is influenced by a complex interplay of cognitive, motivational, socioeconomic, and cultural barriers, as well as misperceptions of individual risk and insufficient understanding of the risk–benefit profile of screening [4].

In this context, there is a growing need for personalised digital tools that support informed decision‐making regarding CRC screening. Digital decision aids (DAs), developed in accordance with evidence‐based medicine principles and user‐centred design, aim to reduce decisional conflict and promote adherence through informed choices [5, 6, 7]. These tools have demonstrated effectiveness in other screening contexts, such as mammography [8], but remain underutilised in Italy for CRC screening.

The DA‐CRC project was conceived to evaluate the impact of a personalised mobile application –PREVenGO—on awareness and uptake of CRC screening among individuals receiving their first screening invitation at age 50. The app was designed to address common participation barriers by providing content tailored to the person's educational level, interactions modulated according to the Locus of Control (LoC) [9], a gamification system to enhance user engagement [10], and an animated educational chatbot.

This pilot study represents a concrete effort to integrate digital technologies—specifically mobile applications—into public health initiatives, in response to evolving epidemiological trends and behavioural shifts in increasingly digital‐savvy, personalisation‐oriented target populations [11, 12, 13, 14].

## 2 Materials and Methods

### 2.1 Study Design

The DA‐CRC study is a single‐centre, randomised controlled trial (RCT) conducted between 1/8/2023 and 31/12/2024. The project is a collaborative initiative between the University of Pavia—Department of Public Health, Experimental and Forensic Medicine—and the Local Health Authority of Brescia (ATS Brescia).

### 2.2 Study Population and Eligibility Criteria

The target population included all residents within the ATS Brescia jurisdiction born between January 1st, 1973, and December 31st, 1974, who were receiving their first official invitation to participate in the regional faecal occult blood test (FOBT)‐based CRC screening programme.

#### 2.2.1 Inclusion Criteria

- Age 50 at the time of recruitment (first invitation in 2023 or 2024).
- Stable residence within one of the municipalities served by ATS Brescia.
- Ownership of a smartphone (Android or iOS).
- Proficiency in the Italian language.
- Provision of informed consent within the PREVenGO app.

#### 2.2.2 Exclusion Criteria

- Failure to provide informed consent.

The target population and the public were not involved in the design, conduction, reporting, or dissemination stages of this research.

### 2.3 Recruitment and Enrolment Procedure

Eligible individuals were invited via the standard screening invitation letter sent by ATS Brescia, which included an additional flyer about the DA‐CRC study and instructions on how to download the PREVenGO mobile application via Quick Response (QR) code. After installing the app, users were guided through the onboarding process by a virtual avatar (Arold, the app mascot), which included the informed consent procedure, GDPR‐compliant privacy information, and completion of the baseline questionnaire (T0).

Participants were then randomised, using an in‐app computer‐generated simple randomisation sequence, into one of two study arms: the intervention group or the control group. Personnel who enrolled or assigned participants had no access to the random allocation sequence.

For the participants to the study, blinding was not feasible for the kind of intervention (Decision Aid) provided by the study and researchers were not blinded.

### 2.4 Intervention Group

Participants randomised to the intervention arm had access to the full‐featured version of the PREVenGO application, which included.

- Interactive educational modules on CRC screening, primary, and secondary prevention.
- Daily microlearning content tailored to educational level (based on the Gulpease Index [15]) and Locus of Control.
- Gamification elements (badges, scores, leaderboards, asynchronous mini‐quizzes, and weekly synchronous quizzes).
- An animated chatbot (Arold) capable of responding to frequently asked questions about prevention and screening.
- Push notifications with motivational content and reminders for both screening and follow‐up questionnaires.

#### 2.4.1 Quizzes Included

- A weekly synchronous quiz with ten questions (five on prevention/screening and five on general knowledge).
- An asynchronous mini‐quiz with three prevention/screening‐related questions.

**FIGURE 1:**
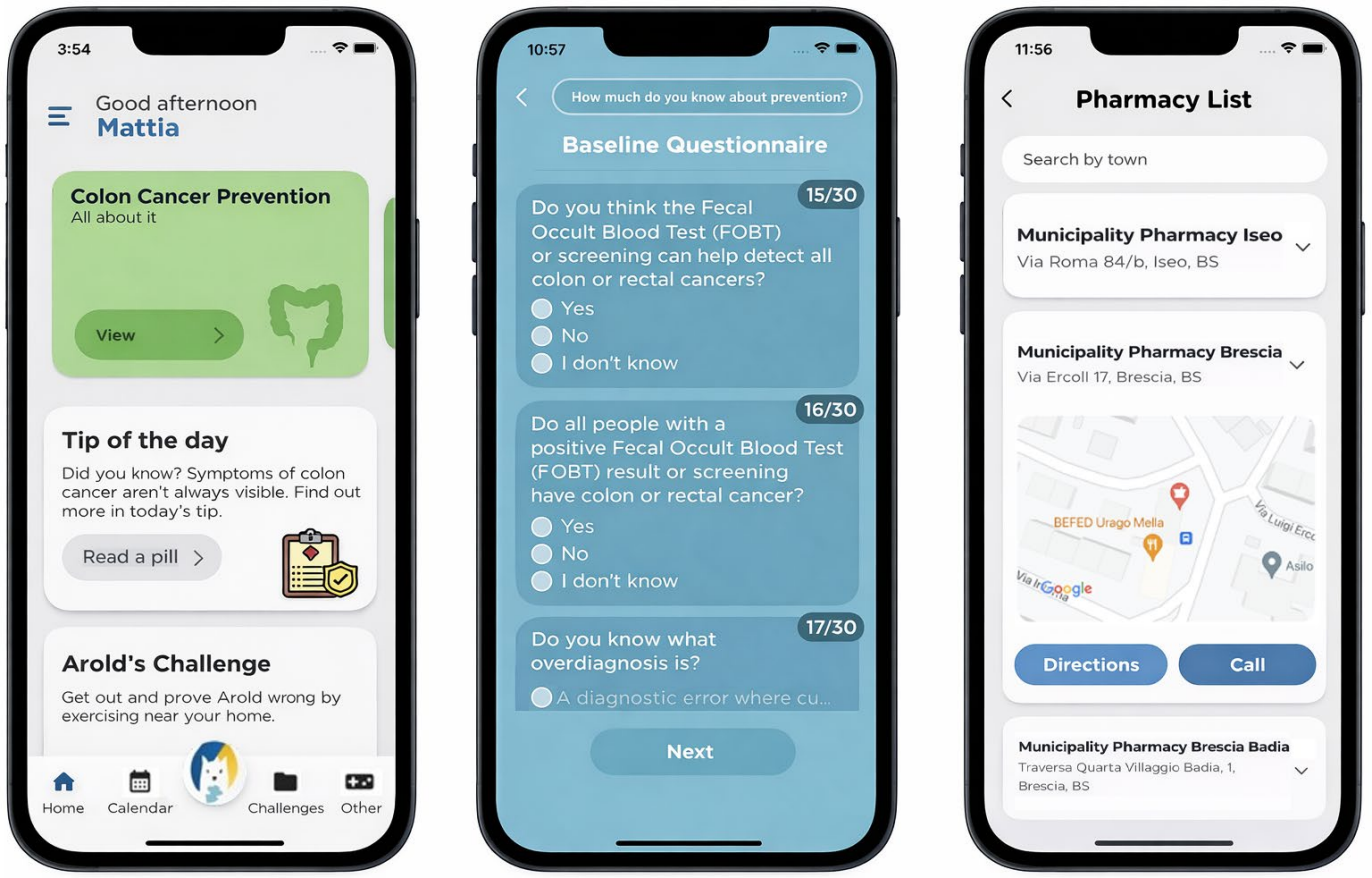
PREVenGO app user interface.

Scoring was proportional to accuracy and speed, with a maximum of ten seconds per question. Figure 1 shows three different screenshots of the PREVenGO mobile application: the left panel displays the home screen as seen by a participant randomised to the intervention group; the central panel shows a couple of baseline questionnaire items; and the right panel presents the map listing the pharmacies distributing the FOBT kit.

### 2.5 Control Group

The control group had access to a “lite” version of the app, containing only static PDF brochures used by ATS Brescia, providing general information on CRC screening and primary prevention. No interactive, gamified, or personalised content was included.

### 2.6 Data Collection Instruments

Data were collected via structured questionnaires.

- The baseline questionnaire (T0) assessed.
  ○ Informed choice, using the three‐dimensional model by Marteau [16, 17], including.
    - Knowledge (minimum score 7/13).
    - Attitude (score > 24).
    - Behavioural intention (actual participation).
  ○ Locus of control (optional), assessed with the validated MHLC questionnaire [18], which includes 18 items.
  ○ Knowledge of primary prevention and healthy behaviours, assessed through 13 multiple‐choice questions. Participants were classified based on correct responses as.
    - Low knowledge (from zero to five correct answers).
    - Moderate knowledge (from six to eight).
    - High knowledge (from nine to thirteen).
  ○ Sociodemographic data, CRC family history, previous screening participation, and smartphone usage.
- Follow‐up questionnaires (T1 at three months and T2 at six months) assessed.
  ○ Informed choice (as above).
  ○ Knowledge of primary prevention and healthy behaviours (same as T0).
  ○ Decisional conflict, user satisfaction, and app acceptability through items related to the decision‐making process and perceived usefulness of the decision aid.

All responses were anonymised using a unique participant ID (PID). ATS Brescia provided pseudonymised data on actual FOBT participation. Only ATS Brescia retained the ability to link PIDs to the individuals invited to the screening programme.

Given the minimal‐risk nature of the experimental trial involving only a mobile application, which does not fall within the field of digital therapy but serves as a decision aid, no harms were defined or assessed in this study. The intervention was not expected to result in any medical or psychological adverse events. For this reason, no stopping guidelines or interim analysis were planned or implemented.

### 2.7 App Development and Data Security

The PREVenGO app was developed in compliance with OWASP (*Open Worldwide Application Security Project*) Mobile Security guidelines, using a serverless, cloud‐based architecture. Data protection and GDPR compliance were ensured through a “privacy by design” framework. The app underwent usability testing with a 25‐user focus group prior to its official release on the Apple App Store and Google Play Store to ensure accessibility, clarity, and user experience.

### 2.8 Sample Size Calculation

Sample size was calculated based on the hypothesis of a minimum clinically significant 10% difference in the proportion of participants making an informed choice [6, 19]. Assuming a statistical power of 90%, a significance level of α = 0.05, and an expected baseline informed choice rate of 50%, a total of 519 participants per group were required. After accounting for an anticipated 20% dropout rate and an estimated 50% adherence rate to colorectal cancer screening in the area covered by ATS Brescia, the final sample size target was set at 2492 participants.

### 2.9 Statistical Analysis

The analysis was restricted to participants who accessed the application, were randomised, and completed the study questionnaire at the scheduled assessment time points. Participants were analysed according to their originally assigned randomisation group (intervention or control). For each assessment time point, a per‐protocol analysis was performed, including only respondents at that specific time point. Missing data from non‐respondents were not imputed.

Individuals who, despite receiving the invitation, did not access the application or did not complete any questionnaire were excluded from the analysis.

An intention‐to‐treat approach involving all invited subjects was not implemented, as the primary aim was to assess the effect of the intervention among participants who were effectively exposed to and engaged with the application. Accordingly, the results reflect the population of respondents at each scheduled assessment.

Categorical variables were summarised using absolute and relative frequencies. Continuous variables were presented as means with standard deviations or medians with interquartile ranges, depending on distribution. Between‐group comparisons were performed using chi‐squared tests for categorical variables, Fisher's exact test when expected frequencies were < 5, and the Wilcoxon‐Mann–Whitney test for ordinal or non‐normally distributed variables. The type II error (β) was set at 0.10, corresponding to a statistical power of 0.90, with a two‐sided significance level (α) at 0.05. Missing data were not expected, since the app has been developed to prevent the users from leaving blank answers in all questionnaires. For the primary and secondary prevention knowledge items, the option “I don't know” was included as a response alternative and was treated as a wrong answer in the analysis. All analyses were performed using STATA MP version 17.0.

## 3 Results

### 3.1 Participation and Randomisation

**FIGURE 2:**
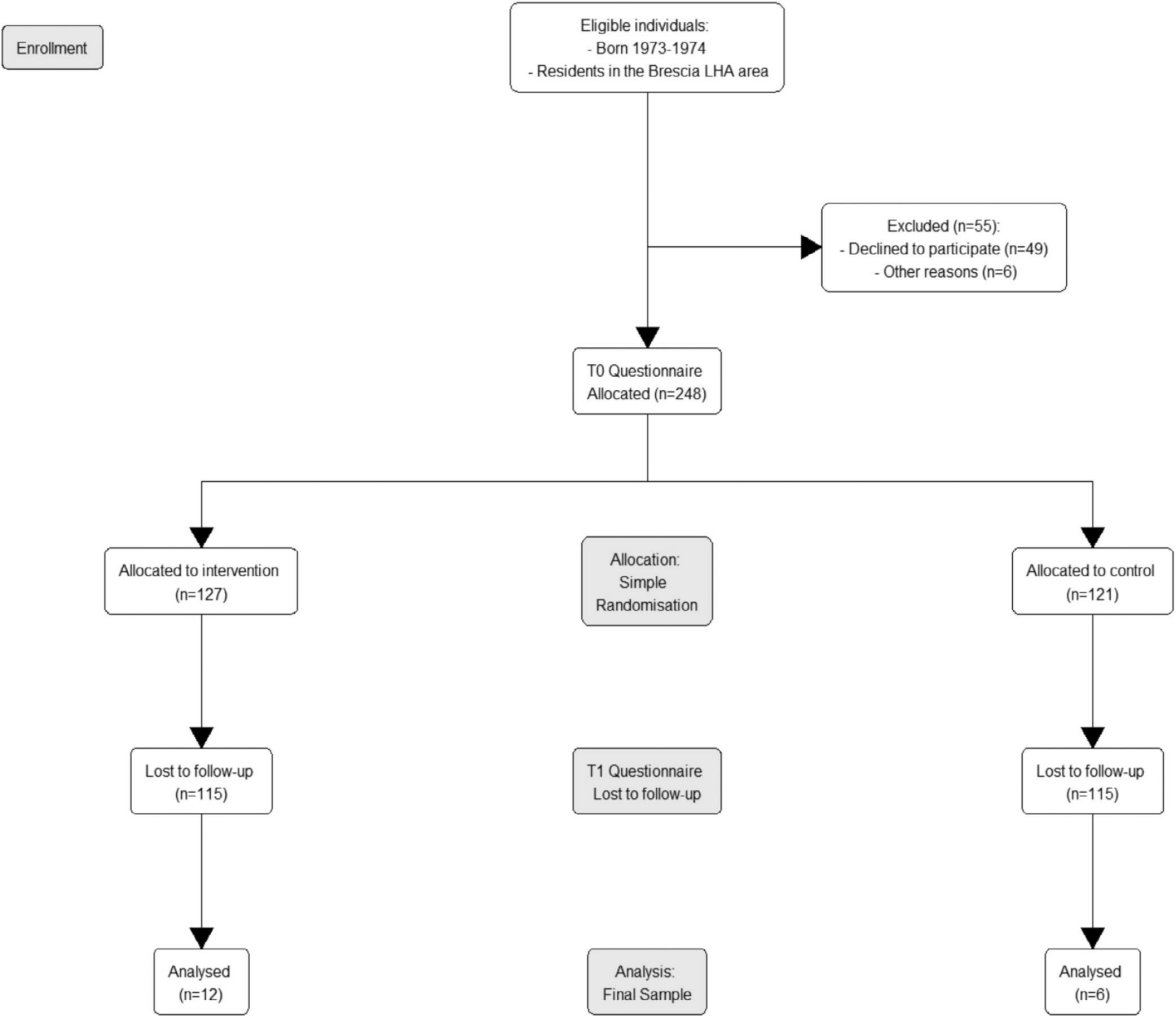
CONSORT flowchart for enrolment and randomisation.

Between May 2023 and December 2024, 303 eligible individuals (born 1973–1974, residents in the ATS Brescia area) downloaded the PREVenGO app. Of these, 248 (81.8%) completed onboarding and provided informed consent; the remaining 55 (18.2%) were excluded due to lack of consent or early dropout during onboarding. Participants were randomised into the control arm (*n* = 121; 48.8%) or the intervention arm (*n* = 127; 51.2%). These details are illustrated in Figure 2.

### 3.2 Baseline Characteristics (T0)

#### 3.2.1 Sociodemographic Characteristics and Data on Internet and Smartphone Usage

Table 1 presents the baseline sociodemographic characteristics and data on internet and smartphone use seeking in the study population, reported both overall and stratified by intervention arm. Overall, 54.4% were male (*n* = 135), 45.6% female (*n* = 113), and 98.8% (*n* = 245) held Italian citizenship. Education levels were distributed as follows: primary school 1.2% (*n* = 3), lower secondary 20.2% (*n* = 50), upper secondary 51.6% (*n* = 128), and university degree or higher 27.0% (*n* = 67). Employment was reported by 91.1% (*n* = 226) of participants; 88.3% (*n* = 219) used smartphones for messaging apps, and 26.6% (*n* = 66) had prior experience with health‐related mobile applications. No statistically significant differences in sociodemographic characteristics were observed between the two arms at baseline (Table 1).

**TABLE 1:** Baseline sociodemographic characteristics and data on internet and smartphone usage.

Variables	Control (*n* = 121)	Intervention (*n* = 127)	Total (*n* = 248)	*p*
Sociodemographic characteristics				
Age	50.5 (0.5)	50.5 (0.5)	50.5 (0.5)	
Sex (Male)	60 (49.6%)	75 (59.1%)	135 (54.4%)	0.135
Citizenship (Italian)	120 (99.2%)	125 (98.4%)	245 (98.8%)	> 0.900^$^
Educational level				0.072^$^
Primary school	3 (2.5%)	0 (0.0%)	3 (1.2%)	
Lower secondary school diploma	18 (14.9%)	32 (25.2%)	50 (20.2%)	
Upper secondary school diploma or equivalent	66 (54.5%)	62 (48.8%)	128 (51.6%)	
University degree or higher	34 (28.1%)	33 (26.0%)	67 (27.0%)	
Employment status (Employed)	108 (89.3%)	118 (92.9%)	226 (91.1%)	0.311
Smartphone usage				
Do you use the Internet to search for health information?				0.458
Never or very rarely	31 (25.6%)	36 (28.3%)	67 (27.0%)	
A few times per month	54 (44.6%)	60 (47.2%)	114 (46.0%)	
At least once a week	15 (12.4%)	14 (11.0%)	29 (11.7%)	
Several times a week	13 (10.7%)	6 (4.7%)	19 (7.7%)	
Daily	8 (6.6%)	11 (8.7%)	19 (7.7%)	
For what purposes do you generally use your smartphone?				
Calls and/or SMS messages (Yes)	96 (79.3%)	105 (82.7%)	201 (81.0%)	0.503
Messaging apps (Yes)	107 (88.4%)	112 (88.2%)	219 (88.3%)	> 0.9
Social Networking (Yes)	69 (57.0%)	56 (44.1%)	125 (50.4%)	0.042**
Games (Yes)	16 (13.2%)	10 (7.9%)	26 (10.5%)	0.169
Browsing the Internet (Yes)	82 (67.8%)	86 (67.7%)	168 (67.7%)	> 0.9
Listening to music, watching movies (Yes)	26 (21.5%)	33 (26.0%)	59 (23.8%)	0.406
Online shopping (Yes)	54 (44.6%)	54 (42.5%)	108 (43.5%)	0.738
Health apps (Yes)	34 (28.1%)	32 (25.2%)	66 (26.6%)	0.605
Others activities (Yes)	46 (38.0%)	31 (24.4%)	77 (31.0%)	0.021*

^$^ Group differences were assessed using Fisher's exact test; * *p*‐value < 0.05.

#### 3.2.2 Knowledge and Awareness

At T0, according to Marteau's three‐dimensional model [17, 18] (knowledge ≥ 7/13, positive attitude, and intention consistent with behaviour), only 43 participants (17.3%) fulfilled all criteria for informed choice. Specifically.

- 19.4% (*n* = 48) achieved sufficient knowledge regarding the FOBT.
- 93.1% (*n* = 231) expressed a positive attitude toward screening.
- 72.2% (*n* = 179) declared definite intention to adhere to the programme.

General knowledge about screening produced moderate results; the most erroneous responses concerned overdiagnosis (*n*. of correct answers: 27, 10,9%), which is the detection of a condition that would never have caused symptoms or harm during a patient's lifetime, leading to unnecessary labelling and potential overtreatment; false positives (*n* = 14; 5,6%), and the absolute mortality benefit of screening (*n* = 22; 8,9%). In contrast, knowledge about primary prevention was high: 69.4% performed excellently (*n* = 172), 28.6% well (*n* = 71), and 2% poorly (*n* = 5). Questions most often answered correctly pertained to physical activity and oncological prevention, while nutrition‐related items showed lower accuracy. No statistically significant differences in knowledge or awareness were observed between the two arms at baseline (T0).

#### 3.2.3 Locus of Control

Out of 121 respondents to the MHLC questionnaire, 62.8% exhibited an internal LoC (*n* = 76), 24.0% (*n* = 29) an external LoC (“others”), and the remainder mixed orientations. These results informed the personalised notification strategy in the intervention arm.

#### 3.2.4 Screening Adherence

Overall FOBT completion was 86.7% (*n* = 215), with 84.3% (*n* = 102) in the control arm and 89.0% (*n* = 113) in the intervention arm. Although the difference did not reach statistical significance (*p* = 0.278), the trend favours the intervention arm.

### 3.3 Three‐Month Follow‐Up (T1)

Only 18 participants completed the T1 questionnaire (6 control, 12 intervention), precluding inferential statistics. Descriptively.

In the control arm, 66.7% (*n* = 4) reached sufficient knowledge levels, compared to 33% (*n* = 4) in the intervention arm.

- Positive attitudes toward screening remained unchanged in both groups.
- “Definite” intention to participate reached 88.9% overall (*n* = 16).
- Primary prevention knowledge remained “excellent” in 72.2% (*n* = 13) and “good” in 27.8% (*n* = 5).

### 3.4 Six‐Month Follow‐Up (T2)

At the latest data extraction (December 2024), only one T2 questionnaire had been completed. Consequently, data on decisional conflict, user satisfaction, and app acceptability is not yet analysable.

## 4 Discussion

The DA‐CRC study yielded two key high‐level findings. First, FOBT screening uptake was remarkably high across both arms (86.7% overall; 84.3% control, 89.0% intervention), suggesting that the enrolled population was already oriented toward health‐protective behaviours. Secondly, and in stark contrast, informed awareness was critically low: only 17% of participants met all criteria of Marteau's framework for an informed decision, with particularly poor understanding of overdiagnosis (6%), false positives, and the absolute mortality benefit of screening. This pattern—high adherence coexisting with low comprehension—points to a model of passive participation rather than active, informed engagement, and raises important ethical questions for organised screening programmes. This study represents one of the first efforts in Italy to evaluate the impact of a mobile‐based digital decision aid (DA) within an organised CRC screening programme; a previous attempt was the 2016 study “*Donna Informata*” [8] on breast cancer screening, involving a web‐based DA that was not optimised for mobile use.

These findings are broadly consistent with the existing literature on decision aids for cancer screening. In a Danish RCT by Gabel et al. (2020), it was demonstrated that a decision aid for CRC screening improved informed choice, especially among individuals with higher educational level—a pattern that mirrors the highly educated, digitally engaged sample of the present study [7]. More broadly, a substantial body of evidence shows that the general population systematically overestimates the benefits of cancer screening and underestimates its harms, including false positives and overdiagnosis [20]. This phenomenon—sometimes described as “popularity without understanding” – has been documented across multiple screening contexts, including breast, cervical, and colorectal cancer, and highlights the critical importance of decision aids as tools for correcting misconceptions rather than for merely increasing uptake [21]. The involvement of general practitioners (GPs) in reinforcing accurate risk communication has been shown to be extremely effective and warrants integration into future iterations of tools like PREVenGO [22, 23].

The DA‐CRC study was conducted in a real‐world public health context and aimed to assess whether a personalised digital tool could enhance informed choice and promote conscious participation among individuals receiving their first screening invitation. This initiative coincided with a critical epidemiological transition, marked by the increasing incidence of early onset colorectal cancer (EOCRC), particularly in Western countries [2, 24]. Such trends call for innovative communication strategies capable of effectively engaging younger segments of the eligible population.

Moreover, locus of control assessment demonstrated that study participants primarily exhibited internal control attribution, a pattern consistent with the high screening adherence observed in our cohort. Paradoxically, despite this internal locus of control, most participants failed to make an informed choice, suggesting that personal health responsibility and positive attitudes toward screening may drive high adherence independently of comprehensive understanding of screening benefits and potential risks.

All these findings suggest that information alone is insufficient to foster meaningful, autonomous decision‐making and reinforce the need to shift from a model of passive adherence to one of active participation in screening programmes in terms of informed choice.

The study also examined the effects of an interactive, gamified app (PREVenGO) compared to a static control version on participants' knowledge and decision‐making. However, the extremely low number of completed follow‐up questionnaires at both time‐points precludes the drawing of robust conclusions about the intervention effectiveness.

Furthermore, the limited sustained use of the app—particularly for follow‐up tasks—points to a broader issue related to engagement. Despite the app user‐centred design and inclusion of gamification features, most participants did not interact with it beyond the initial onboarding phase. Nevertheless, despite this engagement gap, the near‐universal uptake of FOBT (86.7%) suggests that the enrolled population was already intrinsically motivated to undergo screening. Hence, the challenge is not to increase screening adherence per se, but to improve the quality of engagement—specifically, to promote genuine informed choice and critical understanding of screening risks and benefits. Future re‐engagement strategies should therefore focus on improving knowledge acquisition, perhaps through integration with GP consultations and trusted communication channels, rather than simply increasing the frequency of push notifications, which may be perceived as intrusive by a high‐adhering population.

## 5 Limitations

This study has several important limitations. First, the trial was substantially underpowered: temporary compliance‐related delays dramatically reduced the achieved sample size compared with the pre‐specified target (248 vs. 2492), thus limiting statistical power and generalisability. Secondly, follow‐up retention was critically low, with only 18 participants (7.3%) completing the 3‐month questionnaire and a single participant completing the 6‐month assessment; this hindered any meaningful comparison between arms at follow‐up and made secondary outcomes (i.e., decisional conflict, app acceptability, sustained knowledge change) non assessable. In the third place, the enrolled population showed characteristics—i.e., high educational attainment, digital proficiency, and high baseline screening adherence—that are unlikely to represent the broader target population, introducing substantial selection bias. Additionally, the voluntary, opt‐in recruitment via invitation letter and QR code further enriched the sample with individuals who were already predisposed to health‐protective behaviours. This may have limited the generalisability of findings to harder‐to‐reach populations with lower digital literacy or different cultural backgrounds. Finally, blinding of participants was not feasible given the nature of the intervention, as noted in the Methods section. Taken together, these limitations suggest that the present study is best interpreted as a feasibility and proof‐of‐concept assessment; adequately powered multisite trials with broader recruitment strategies are needed to draw definitive conclusions.

Nonetheless, the DA‐CRC experience provides valuable insights into the feasibility, acceptability, and scalability of digital DAs in public screening contexts. Notably, the app's modular design and adaptable framework can be translated to other screening programmes (e.g., breast, cervical) and across broader domains of primary and secondary prevention. As public health increasingly embraces digitalisation, tools like PREVenGO, when rigorously co‐designed with end users and embedded within multichannel strategies, offer a promising path to improve informed engagement and shared decision‐making.

## 6 Conclusions

In conclusion, the DA‐CRC study highlights that information alone is not enough to support meaningful, autonomous decision‐making, underscoring the need to move from passive compliance toward active engagement in screening programmes. The study revealed a striking contrast between the high uptake of the screening test and the low prevalence of informed choices, thus having important healthcare policy implications for public health interventions.

Furthermore, the study highlights how integration of digital decision aids into cancer screening pathways is both technically and ethically feasible; however, it also shows that digital tools, if not adequately supported by active engagement strategies and accessible design, may fall short of their potential. Future research should prioritise hybrid models that combine digital innovation with personalised communication and real‐world support systems, thereby addressing the persistent gap between participation and informed awareness in cancer prevention.

## Author Contributions

**Alessandro Summer:** conceptualization, investigation, writing – original draft, writing – review and editing, data curation. **Paolo La Torraca Vittori:** conceptualization, investigation, writing – original draft, writing – review and editing, data curation. **Alessandro Incremona:** conceptualization, investigation, writing – original draft, data curation, writing – review and editing. **Francesco Brucchi:** conceptualization, investigation, writing – original draft, writing – review and editing, data curation. **Paola Ghidini:** writing – review and editing, data curation. **Maria Vizzardi:** writing – review and editing, data curation. **Mattia Rogledi:** conceptualization, investigation, writing – original draft, data curation, writing – review and editing. **Michela Bregoli:** writing – review and editing, data curation. **Enri Hoxha:** writing – review and editing, data curation. **Cristina Montomoli:** conceptualization, writing – original draft, funding acquisition, writing – review and editing, methodology, validation, formal analysis, project administration, supervision, data curation. **Asia Filosa:** conceptualization, methodology, data curation, formal analysis, writing – review and editing, writing – original draft, validation. **Giovanni Marazza:** data curation, writing – review and editing, supervision. **Manuela Anelli:** conceptualization, writing – original draft, methodology, validation, writing – review and editing, data curation, formal analysis. **Alessandra Gorini:** conceptualization, methodology, writing – review and editing, validation. **Mattia Celebrin:** conceptualization, writing – original draft, writing – review and editing, data curation, investigation.

## Funding

The study had been developed within the framework of the Lombardy Regional Prevention Plan 2021–2025, specifically the *PPO2 (“Active Communities”)* and *PL14 (“Cancer Screening”)* programmes. The study was commissioned and funded by the Local Health Authority of Brescia (Agenzia di Tutela della Salute di Brescia ‐ ATS Brescia). People eligible for enrolment in this study were those residing in the area of competence of ATS Brescia. ATS Brescia was the only organisation involved in the study capable of associating PID identifiers to an individual tax code to gather screening adherence data. ATS Brescia contributed to the project by providing the informational material on colorectal screening, which was then revised and adapted for use in the study application. However, it was not involved in any of the writing of the study protocol or the data analysis.

## Ethics Statement

The study protocol was approved by the reference ethical committee of Brescia on 26th January 2023, protocol n° NP5686.

## Conflicts of Interest

M.B., P.G., E.H., G.M. and M.V. are affiliated with ATS Brescia, which provided funding but had no role in the design of the study, analysis, or interpretation of the data. The other authors declare that they have no involvement with any organisation or entity with any financial interest or non‐financial interest in the subject matter or materials discussed in this manuscript.

## Data Availability

The data that support the findings of this study are available from the corresponding author upon reasonable request.
