# Correction to ‘A Personalised and Gamified Digital Decision Aid for Colorectal Cancer Screening: A Randomised Controlled Pilot Study’

**DOI:** 10.1002/cam4.72069

**Published:** 2026-06-21

M. Celebrin , A. Filosa , M. Anelli , et al., “A Personalised and Gamified Digital Decision Aid for Colorectal Cancer Screening: A Randomised Controlled Pilot Study,” Cancer Medicine 15, no. 6 (2026): e71991, 10.1002/cam4.71991.42216438 PMC13240130

The authors' first and last names were swapped and have been updated in the author list, Correspondence section, and Author Contributions section.

The online version of this article has been corrected accordingly.

We apologize for this error.

